# Nanoscale modeling of dynamically tunable planar optical absorbers utilizing InAs and InSb in metal-oxide-semiconductor–metal configurations

**DOI:** 10.1186/s11671-023-03879-5

**Published:** 2023-08-11

**Authors:** Kirtan P. Dixit, Don A. Gregory

**Affiliations:** Department of Physics and Astronomy, The University of Alabama in Huntsville, 301 Sparkman Drive, Huntsville, AL 35899 USA

**Keywords:** Tunable optical absorbers, Spectrally selective optical absorption, InAs, InSb

## Abstract

The attainment of dynamic tunability in spectrally selective optical absorption has been a longstanding objective in modern optics. Typically, Fabry–Perot resonators comprising metal and semiconductor thin films have been employed for spectrally selective light absorption. In such resonators, the resonance wavelength can be altered via structural modifications. The research has progressed further with the advent of specialized patterning of thin films and the utilization of metasurfaces. Nonetheless, achieving dynamic tuning of the absorption wavelength without altering the geometry of the thin film or without resorting to lithographic fabrication still poses a challenge. In this study, the incorporation of a metal-oxide-semiconductor (MOS) architecture into the Fabry–Perot nanocavity is shown to yield dynamic spectral tuning in a perfect narrowband light absorber within the visible range. Such spectral tuning is achieved using n-type-doped indium antimonide and n-type-doped indium arsenide as semiconductors in a MOS-type structure. These semiconductors offer significant tuning of their optical properties via electrically induced carrier accumulation. The planar structure of the absorber models presented facilitates simple thin-film fabrication. With judicious material selection and appropriate bias voltage, a spectral shift of 47 nm can be achieved within the visible range, thus producing a discernible color change.

## Introduction

Recently, there has been significant progress in the development of spectrally selective optical absorber and modulators, including patterned and unpatterned thin-film structures. These structures find use in a wide range of applications such as biological and chemical sensing [1–4], structural color printing [5, 6], solar energy harvesting [7, 8], and color filters [9–11]. However, the incorporation of tunability into these structures has presented a significant challenge. Efficient modification of the refractive index of the materials employed in the design, as well as the enhancement of light–matter interaction, has remained constant areas of focus in pursuit of tunability. In recent times, researchers have adopted a strategy that involves utilizing an epsilon-near-zero (ENZ) semiconductor as the active layer for developing dynamically tunable optical absorbers/modulators [12–27]. Transparent conductive oxides (TCO) and doped semiconductors such as indium tin oxide (ITO) [12, 13, 15, 16, 20, 21, 23–25], zinc oxide (ZnO) [28], aluminum-doped zinc oxide (AZO) [17], indium oxide ($\text {In}_{2}\text {O}_{3}$) [18], cadmium oxide (CdO) [29], indium antimony (InSb) [14, 26], and indium arsenide (InAs) [27, 30] show excellent light modulation performance based on the ENZ effect. The fundamental concept underlying the operation of many of these modulators revolves around leveraging the influence of an external potential on the majority charge carriers within a heavily doped ENZ semiconductor. This external potential subsequently impacts the plasma frequency ($\omega _p$) of the semiconductor.

The effect of external potential on the plasma frequency is a direct prediction of the classic Drude model for light–matter interaction [31, 32], which describes the relationship between the plasma frequency, relative permittivity ($\epsilon _r$), and the scattering constant ($\gamma$) of a material, as well as the incoming light frequency ($\omega$). As per Eq. (1) (from the Drude model), the plasma frequency is a function of electron concentration (N) and is dependent on various parameters such as the electron’s effective mass ($m^*$), charge (q), and vacuum permittivity ($\epsilon _0$). The change in $\omega _p$ in turn affects the $\epsilon _r$ of the doped semiconductor, through the relationship:

1 $$\begin{aligned} \epsilon _r(\omega ) &= \epsilon _{\infty }+\frac{\omega _p^2}{i\gamma \omega -\omega ^2},\nonumber \\ \text {where}\ \omega _p&= \sqrt{\frac{\text {N}\text {q}^2}{m^* \epsilon _0}} \end{aligned}$$

The $\epsilon _r(\omega )$ of a material is a complex function that can be expressed as $\epsilon _r(\omega ) = \epsilon ^{'}(\omega )+\textit{i}\epsilon ^{''}(\omega )$, where $\epsilon ^{'}(\omega )$ and $\epsilon ^{''} (\omega )$ are real and imaginary components, respectively. The equations for $\epsilon ^{'}(\omega )$ and $\epsilon ^{''} (\omega )$ can be written as:

2 $$\begin{aligned} \epsilon ^{'}(\omega )&= \epsilon _{\infty }-\frac{\omega _p^2}{\omega ^2+\gamma ^2},\nonumber \\ \epsilon ^{''}(\omega )&= \frac{\gamma \omega _p^2}{\omega (\omega ^2+\gamma ^2)} \end{aligned}$$

The $\epsilon _r$ also determines the effective refractive index ($n_{eff}$) of a material, as defined by $n_{eff} (\omega )=\sqrt{(\epsilon _r (\omega ) )}$. This relationship can be written as $\text {n}(\omega )+\textit{i}\kappa (\omega )=\sqrt{(\epsilon ^{'} (\omega )+\textit{i}\epsilon ^{''} (\omega ) )}$, where n and $\kappa$ represent the real and imaginary components of the effective refractive index of the material. The real component, n, governs the dispersion of light in the material, while the imaginary component, $\kappa$, signifies the absorption of light. From Eq. (2), it can be seen that for a given value of plasma frequency ($\omega _p$), a high-frequency permittivity ($\epsilon _{\infty }$), and a scattering constant ($\gamma$), the value of $\epsilon ^{'}(\omega )$ becomes zero at a specific frequency ($\omega$). This frequency is known as the ENZ frequency and can be obtained by rearranging the equation for $\epsilon ^{'} (\omega )$ and setting it equal to zero.

3 $$\begin{aligned} \omega _{ENZ}=\sqrt{\frac{\omega ^2_{p}-\epsilon _{\infty }\gamma ^2}{\epsilon _{\infty }}},\ \lambda _{ENZ}=\frac{2\pi c}{\omega _{ENZ}} \end{aligned}$$

Therefore, the ENZ frequency ($\omega _{ENZ}$) can be considered as a transition point where the real component of the permittivity ($\epsilon ^{'}$) changes from positive to negative [33, 34]. This transition also affects the effective refractive index ($n_{eff}$), as a negative $\epsilon _r$ results in an increase in the imaginary component ($\kappa$), thereby increasing the absorption in the material for all frequencies below $\omega _{ENZ}$. Although the phenomenon of epsilon-near-zero (ENZ) materials is intriguing, it is important to note that free-standing ENZ materials do not exhibit significantly enhanced optical absorption when subjected to normal incident light at frequencies lower than $\omega _{ENZ}$. This lack of enhancement arises from the absence of an electric field component perpendicular to the interface for normal incident light [20]. Therefore, to achieve dynamic tunability in optical modulators and improve absorption, the utilization of ENZ materials alone is insufficient. It is imperative to design the modulator in a manner that enhances the strength of the interaction between light and matter. In essence, both the selection of an appropriate ENZ material and the careful design of the modulator are necessary to achieve the desired enhanced light–matter interaction strength.

In the majority of the tunable electro-optical modulator designs based on the epsilon-near-zero (ENZ) effect, the ENZ effect is primarily confined to an ultrathin layer at the interface between the dielectric and semiconductor materials. As a result, the modulation performance relies on the strength of the interaction between light and the semiconductor, specifically the degree to which light is concentrated within the semiconductor sublayer, thereby enhancing absorption through the ENZ effect [12, 13, 16, 17, 20, 24–27].

In ENZ material-based tunable optical devices, the modification of the transition frequency ($\omega _{ENZ}$) of the material is crucial in changing its optical behavior. This is achieved by altering the material’s plasma frequency ($\omega _p$), which is highly dependent on the electron concentration (N). Thus, generating a charge gradient within a doped semiconductor (with an applied potential bias) is one of the methods to alter $\omega _p$ throughout the material layer [12, 13, 16, 17, 20, 23–27]. The majority of electrically tunable optical modulators based on epsilon-near-zero (ENZ) materials are meticulously designed to facilitate the coupling between the ENZ region induced by the applied potential and the plasmon resonance mode of the structure. This deliberate coupling is aimed at enhancing the optical absorption capabilities of the modulator [16, 20, 24, 27]. However, such structures do require complex lithography patterning.

**Fig. 1:**
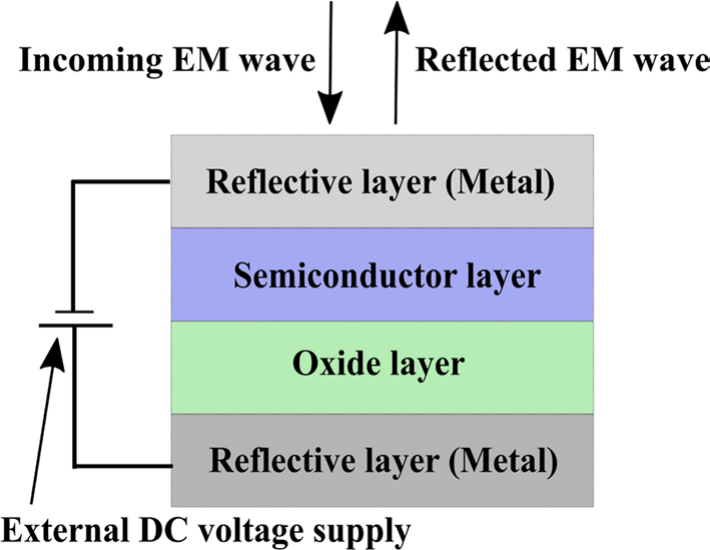
Schematic structure of metal-oxide-semiconductor–metal Fabry–Perot nanocavity

In contrast to other designs of dynamically tunable modulators that primarily focus on enhancing absorption within specific wavelength bands or plasmon modes, the proposed research showcases the optical tuning capabilities of a Fabry–Perot nanocavity. This nanocavity is constructed using a metal-oxide-semiconductor–metal (MOSM) structure (see Fig. 1) and aims to achieve spectral tuning of a Fabry–Perot resonance within the visible range. The key feature of this design lies in the utilization of an epsilon-near-zero (ENZ) semiconductor as the semiconductor layer within the MOSM structure. This incorporation enables dynamic control and manipulation of the refractive index within the semiconductor sublayer, facilitating precise tuning of the nanocavity’s optical properties.

In general, in a nanocavity composed of two reflecting metal mirrors separated by a dielectric spacer, the propagation round-trip phase delay in the spacer is $\phi _{s}$ = 4$\pi$n*d*/$\lambda$, and the reflection phase change with top and metal boundaries is $\phi _{ms}$ (metal–semiconductor) and $\phi _{om}$ (oxide–metal). For the given MOSM structure, *d* is the thickness of the spacer ($d_{oxide} + d_{semiconductor}$), n is the effective refractive index of the spacer, and $\lambda$ is the effective wavelength. The presented study follows the idea of tuning the spectral response of nanocavity by manipulating the refractive index of the semiconductor sublayer using an external potential [26, 28]. Further in the article, the effect of this manipulation on the resonance is elaborated upon in detail through the application of the transfer matrix method.

In this research, the concept of manipulating the effective refractive index of a doped semiconductor is thoroughly explored using a combination of electronic and optical descriptions. The practical applications of this concept are illustrated in the design and operation of dynamically tunable FPN models composed of the MOSM architecture. To begin, we developed a modeling methodology employing the transfer matrix method (TMM) for MOSM configurations [10, 11]. The aim was to identify suitable metals and oxide materials to be used in conjunction with n-InSb and n-InAs for achieving narrow-band perfect absorption within the visible range. Based on the modeling results, the MOSM configurations featuring gold (Au), aluminum (Al), titanium dioxide (${\text {TiO}}_2$), n-InSb, and n-InAs were designed as follows: (1) Au (100 nm)–${\text {TiO}}_2$ (10 nm)–(n-InSb) (20 nm)–Au (17 nm), (2) Al (100 nm)–${\text {TiO}}_2$ (12 nm)–(n-InSb) (20 nm)–Al (8 nm), (3) Au (100 nm)–${\text {TiO}}_2$ (10 nm)–(n-InAs) (15 nm)–Au (15 nm), and (4) Al (100 nm)–${\text {TiO}}_2$ (15 nm)–(n-InAs) (20 nm)–Al (8 nm).

## Theory and absorber structure

The functioning of the dynamically tunable nanocavity is based on a synergistic interaction between electrical and optical processes that take place within the semiconductor layer, particularly at the interface between the oxide and semiconductor materials [12, 13, 16–22, 25–27].

### Electronic process

The structure depicted in Fig. 1 closely resembles the metal-oxide-semiconductor capacitor (MOScap) architecture, with the top metal layer serving solely as an electrical contact. The metal-oxide-semiconductor (MOS) structure is widely utilized for its ability to control charge concentration location inside the semiconductor layer to within a few nanometers. The effect of an applied electric field on charge concentration at the oxide-semiconductor interface has been exploited for a range of practical applications, such as in power electronics [35], sensing devices [36, 37], and memory devices [38, 39]. The nanocavity presented in this work holds the potential to become a new optical application of the MOS structure. The MOScap design is similar to a parallel plate capacitor, with the metal and semiconductor layers serving as the capacitive plates and the oxide serving as the dielectric medium. Upon application of a positive bias to the metal layer (referred to as the gate), the majority charge carriers (electrons) of the n-type doped semiconductor layer accumulate at the oxide-semiconductor interface. This is known as the accumulation mode of a MOScap [40].

The charge density in the accumulation mode is dependent on the electric potential distribution across the semiconductor layer. Poisson’s equation provides a means of evaluating potential differences between different points in such systems. The solution of Poisson’s equation in the surface region of the semiconductor (such as n-InSb or n-InAs in this study) near the oxide-semiconductor interface yields a relationship between the surface potential ($\psi _s$), charge (q), and electric field (*E*). In the case of an n-type semiconductor, Poisson’s equation can be expressed as follows:

4 $$\begin{aligned} \frac{{d}^2\psi }{{d}{x}^2}&= -\frac{{d}{E}}{{d}{x}} \nonumber \\ &= -\frac{\text {q}}{\epsilon _{semi.}} {[p(x)-n(x)+N_{d}(x)-N_{a}(x)]} \end{aligned}$$

where $\psi$ is the electrostatic potential, $\epsilon _{semi.}$ is the permittivity of the semiconductor material, *p*(*x*) and *n*(*x*) are the densities of holes and electrons at any position *x* within the semiconductor, respectively, $N_{d} (x)$ is the donor concentration, and $N_{a} (x)$ is the acceptor concentration. Many electrostatic parameters in the semiconductor layer can be expressed in terms of this electrostatic potential. With no applied field, a uniformly doped n-type or p-type semiconductor is charge neutral—the right-hand side of Eq. (4) is zero, and the potential is constant throughout the semiconductor sample.

**Fig. 2:**
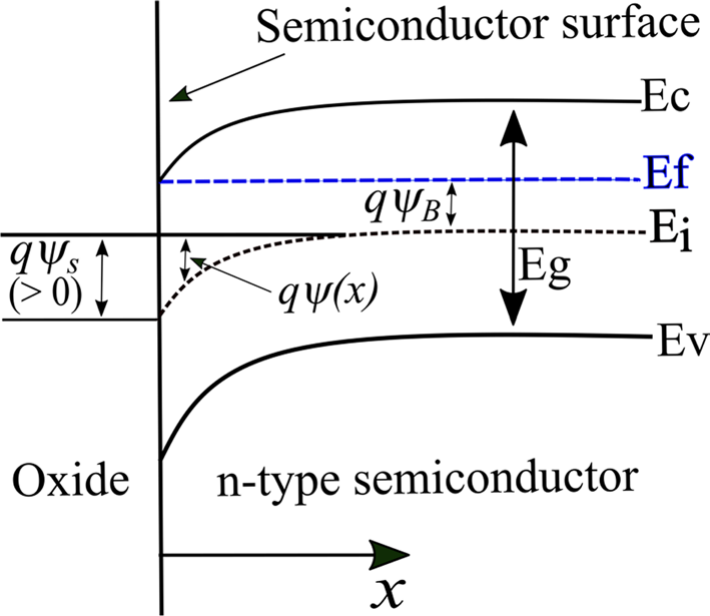
Energy-band diagram near the semiconductor surface of a MOS device with n-type semiconductor. The band bending potential is defined as positive when the bands bend downward in accumulation mode

To illustrate the impact of the electrostatic potential ($\psi$), the energy band diagram serves as a valuable tool. The potential ($\psi$) is typically defined relative to the intrinsic Fermi level and can thus be represented as $\psi _i$. In n-type doped semiconductors, the Fermi potential ($\psi _f$) and donor concentration ($N_d$) are also significant electronic parameters. The difference between $\psi _i$ and $\psi _f$ in the band diagram is referred to as $\psi _B$, as depicted in Fig. 2.

The relationship between $\psi _B$ and the doping concentration (or the ionized dopant concentration for incomplete ionization) is established through the following:

5 $$\begin{aligned} \psi _{B}=\psi _{f}-\psi _{i}=\frac{kT}{q}\text {ln}\left( \frac{N_{d}}{n_{i}}\right) \end{aligned}$$

In this equation, *k* represents the Boltzmann constant, *T* represents the ambient absolute temperature, and ${n}_i$ represents the intrinsic carrier density of the semiconductor material. It is worth noting that Eq. (5) is based on the principle of charge neutrality and is valid only when the local net charge density, being the sum of the mobile and ionized dopant charge densities, is zero, in the right-hand side of Eq. (4). In instances where a net charge exists (due to a discrepancy between the mobile and fixed charge densities) and band bending (spatial variations of $\psi _i$ under applied potential) occurs, the carrier densities in relation to the electrostatic potential can be expressed as follows:

6 $$\begin{aligned} n = n_{i}e^{q(\psi _{i}-\psi _{f})/{kT}},\ p = n_{i}e^{q(\psi _{f}-\psi _{i})/{kT}} \end{aligned}$$

These relations are known as Boltzmann’s relations and are applicable to both n-type and p-type semiconductors in thermal equilibrium.

As illustrated in Fig. 2, the potential $\psi (x) = \psi _i (x) - \psi _i (x=\infty )$ represents the degree of band bending at position *x*, with $x=0$ being the oxide-semiconductor interface and $\psi _i (x=\infty )$ being the intrinsic potential beyond the interface. A positive value of $\psi (x)$ indicates that the band is bent downward. The boundary conditions are defined as $\psi =0$ away from the interface and $\psi =\psi (0)=\psi _s$ at the interface, where accumulation occurs when $\psi _s >0$. The electron and hole concentrations at any given point *x* within the semiconductor can be expressed as a function of $\psi (x)$ using $\psi (x)=\psi _i (x) - \psi _i (x=\infty )$ in Eq. (5). The expressions for *n*(*x*) and *p*(*x*) in terms of $\psi (x)$ are as follows:

7 $$\begin{aligned} n(x) = n_{i}e^{q(\psi {(x)}-\psi _{B})/{kT}}= n_{0}e^{q\psi (x)/{kT}} \end{aligned}$$

8 $$\begin{aligned} p(x) = n_{i}e^{q(\psi _{B}-\psi {(x)})/{kT}}= \frac{n^2_{i}}{n_{0}}e^{-q\psi (x)/{kT}} \end{aligned}$$

where $n_0$ represents the majority carrier density of electrons at $x=\infty$ and ${n}_i^2/n_0$ represents the minority carrier density of holes at $x=\infty$, respectively.

Therefore, the solution of Poisson’s equation determines the distribution of electrostatic potential at every point *x* within the semiconductor layer of the MOScap, which, in turn, defines the electron and hole density distributions across the semiconductor (as described in Eqs. 7 and 8) at the applied gate potential. In this study, the distributions of electrostatic potential and majority carriers for four systems comprising n-InSb or n-InAs semiconductor layers and ${\text {TiO}}_2$ oxide layers have been calculated using the CHARGE solver of the Ansys LUMERICAL software [41]. The solver iteratively solves Poisson’s equation for the given MOScap system. To achieve precise results, it is important to have a fine enough mesh setting in the solver to sample the carrier distribution curves accurately. The results of this analysis are presented and discussed in detail later.

### Absorber structure and optical process

**Fig. 3:**
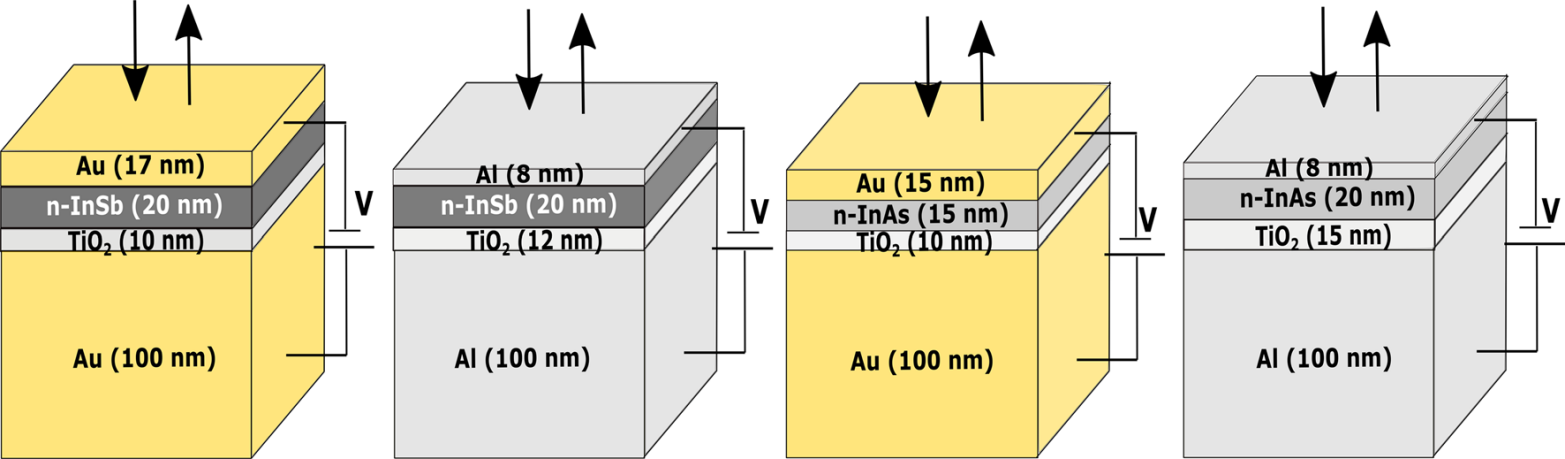
Schematic diagram of proposed absorber structures with the thickness of each layer in nm. The structures (from the left) are (1) Au (100 nm)–${\text {TiO}}_2$ (10 nm)–(n-InSb) (20 nm)–Au (17 nm), (2) Al (100 nm)–${\text {TiO}}_2$ (12 nm)–(n-InSb) (20 nm)–Al (8 nm), (3) Au (100 nm)–${\text {TiO}}_2$ (10 nm)–(n-InAs) (15 nm)–Au (15 nm), and (4) Al (100 nm)–${\text {TiO}}_2$ (15 nm)–(n-InAs) (20 nm)–Al (8 nm)

The proposed FPN (Fig. 1) consists of reflective metal layers and a semiconductor with an oxide layer acting as the medium. The thickness of the medium determines the resonance wavelength, which is found to be in the visible range for the four structures presented in Fig. 3. The use of ${\text {TiO}}_2$ as the oxide layer increases the dielectric breakdown voltage (due to its high dielectric constant) [42].

**Fig. 4:**
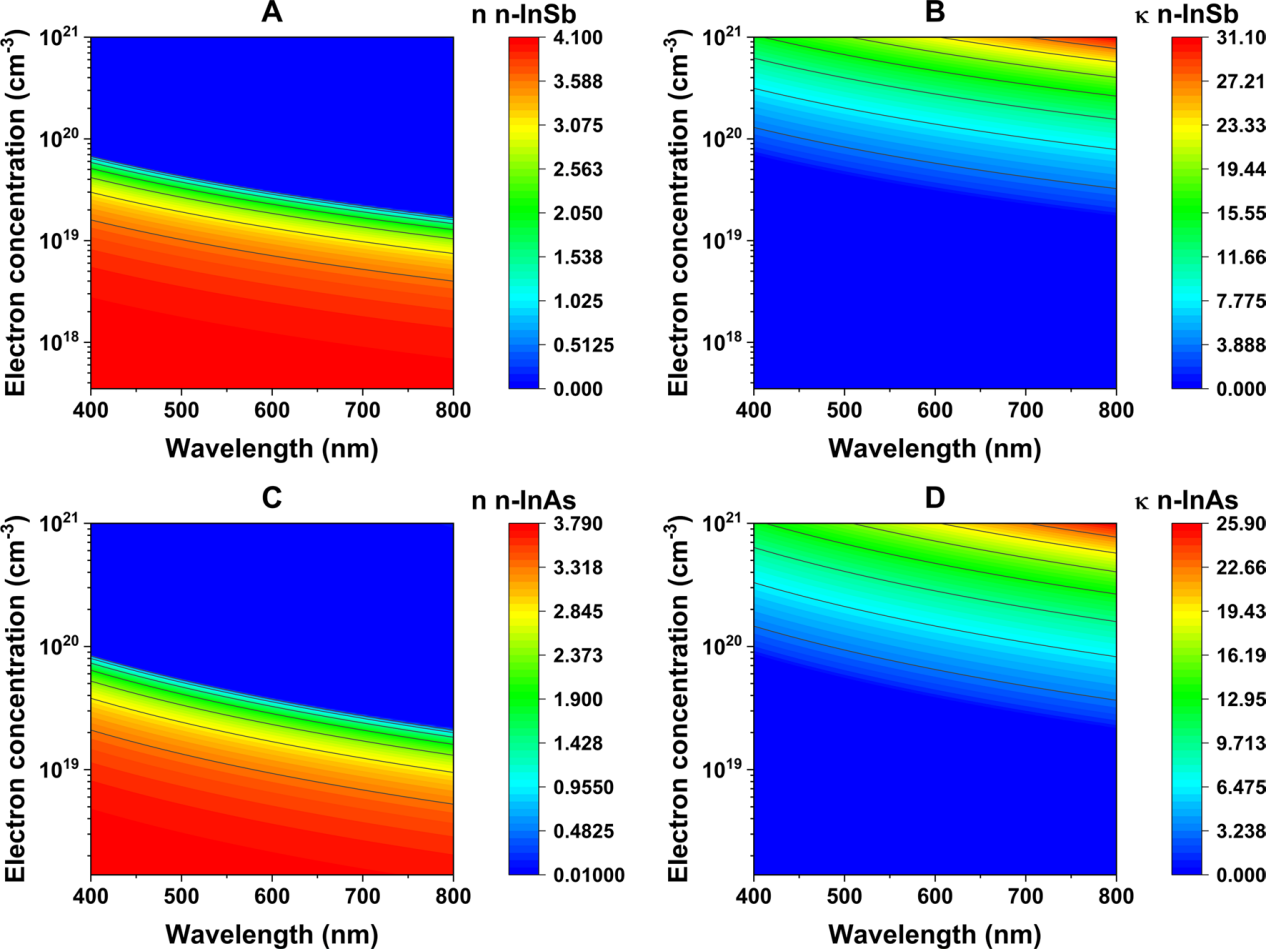
Calculated (**A**) real and (**B**) imaginary parts of the effective refractive index of n-InSb for N$_1 = 3.5 \times 10^{17}$/cm$^3$ to N$_2 = 1 \times 10^{21}$/cm$^3$, (**C**) real and (**D**) imaginary parts of the effective refractive index of n-InAs for N$_1= 1.4 \times 10^{18}$/cm$^3$ to N$_2 = 1 \times 10^{21}$/cm$^3$

The optical behavior of the semiconductors n-InSb and n-InAs is crucial in understanding the optical response to an external electric potential. Carrier concentrations of ${\text {N}}_1^{\text {(n-InSb)}} = 3.5\times 10^{17}/\text {cm}^3$ and ${\text {N}}_1^{\text {(n-InAs)}} = 1.4\times 10^{18}/\text {cm}^3$ were used in the simulation. To understand the effects of free carrier concentration density on the effective refractive index, the results using the calculated parameters n$(\omega )$ and $\kappa (\omega )$ from the Drude model for both semiconductors, with N values $3.5\times 10^{17}/\text {cm}^3$ to $10^{21}/\text {cm}^3$, and from $1.4\times 10^{18}/\text {cm}^3$ to $10^{21}/\text {cm}^3$, are presented in Fig. 4. One should note that, due to the limitation of solid solubility of the dopant, one cannot physically dope a semiconductor such as InAs with a concentration higher than $10^{19}/\text {cm}^3$ [30]. However, a temporary electron concentration of the order of $10^{21}$–$10^{22}/\text {cm}^3$ can be achieved within a sublayer of the semiconductor using the effects of external electric potential on certain types of device architectures as discussed in this article.

**Fig. 5:**
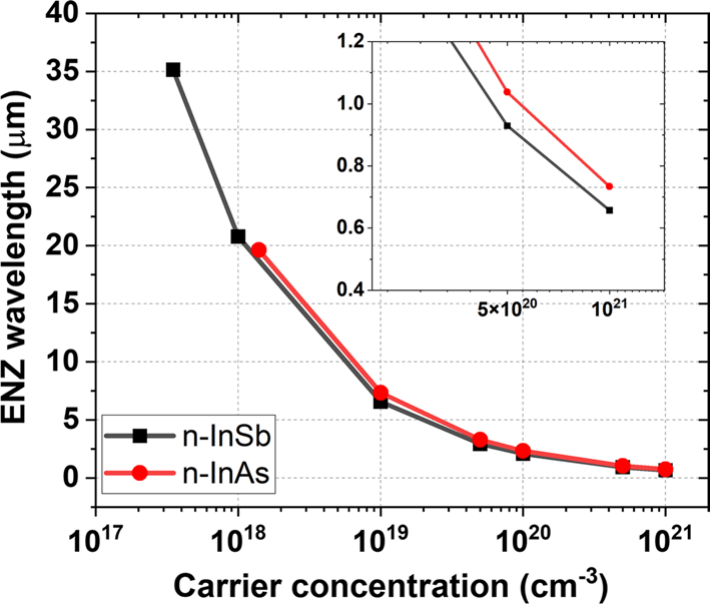
Epsilon near-zero (ENZ) wavelength as a function of carrier concentration for n-InSb and n-InAs

As can be observed in Fig. 5, with increasing carrier concentration, the $\lambda _{ENZ}$ shifts from the far-infrared (35.14 $\upmu$m) to the visible range (0.657 $\upmu$m) for n-InSb, and from the mid-infrared (19.61 $\upmu$m) to the visible spectrum (0.734 $\upmu$m) for n-InAs. The parameters used to calculate the n$(\omega )$ and $\kappa (\omega )$ using the Drude model are listed in Table 1.

**Table 1:** Measured optical properties of n-InSb and n-InAs [43]

Material	$\epsilon _{\infty }$	$m^{*}$	$\gamma (\text {rad/s})$
n-InSb	16.8	$0.023m^{(\textrm{a})}_{e}$	$2.12\times 10^{12}$
n-InAs	14.6	$0.033m^{(\textrm{a})}_{e}$	$2.63\times 10^{12}$

$^{(\textrm{a})}m_{e}= 9.1\times 10^{-31}$ kg (electron rest mass)

Using Figs. 4 and 5, two ranges of wavelength ($\lambda$) can be observed: 1) $\lambda <\lambda _{ENZ}$ and 2) $\lambda >\lambda _{ENZ}$. For wavelengths that fall under the first case, it is evident that the real part of the refractive index plays a dominant role in determining the optical behavior, leading the material to act like a dielectric. On the other hand, for wavelengths that follow the second case, the imaginary part of the effective refractive index becomes dominant as the real part of the permittivity becomes negative. This leads the material to behave optically like a metal.

In the proposed MOSM structures, when an external potential is applied, it follows the electrostatic potential distribution described in the earlier section. This results in a noticeable charge gradient ($\Delta$N) in the n-InSb and n-InAs semiconductors due to the majority charge distribution generated by the applied potential. As a result, n-InSb and n-InAs show different values of $\lambda _{ENZ}$ across the layer when an external potential is applied.

To actively modify the optical performance of the proposed FPNs, it is crucial to manipulate the effective refractive index of the n-InSb or n-InAs layers through changes in the electron concentration gradient.

The transfer matrix method (TMM) is a theoretical tool that can uncover the overall optical effect of the refractive index gradient in the proposed optical cavities, resulting in a global relationship between the electric-field vectors of the incident, reflected, and transmitted electromagnetic waves [44]. Mathematically, the TMM is a $2\times 2$ matrix that incorporates information about all partial reflections and transmissions within a multilayer thin-film structure. The TMM uses propagation and transmission matrices to calculate a final transfer matrix (*M*), and the reflectance at a specific wavelength can be determined by two of the transfer matrix elements. The propagation ($p_m$) and transmission ($D_m$) matrices are components of the TMM and can be written as:

9 $$\begin{aligned} P_m &= \begin{bmatrix} exp(\textit{i}\phi _{m}) &{} 0\\ 0 &{} exp(-\textit{i}\phi _{m}) \end{bmatrix},\nonumber \\{} & {} \text {where}\ \phi _{m}=\frac{2\pi (\text {n}_{m}-\textit{i}\kappa _{m})d_{m}}{\lambda cos \theta _{t}}. \end{aligned}$$

For TE polarization,

10 $$\begin{aligned} D_m = \begin{bmatrix} 1 &{} 1\\ (\text {n}_{m}-\textit{i}\kappa _{m})cos\theta _{m} &{} -(\text {n}_{m}-\textit{i}\kappa _{m})cos\theta _{m} \end{bmatrix} \end{aligned}$$

and for TM polarization,

11 $$\begin{aligned} D_m = \begin{bmatrix} cos\theta _{m} &{} cos\theta _{m}\\ (\text {n}_{m}-\textit{i}\kappa _{m}) &{} -(\text {n}_{m}-\textit{i}\kappa _{m}) \end{bmatrix} \end{aligned}$$

Here, *m* = 0, 1, 2, 3, 4 represents air, the top metal layer (Au or Al), the semiconductor layer (n-InSb or n-InAs), the oxide layer (${\text {TiO}}_2$), and the bottom metal layer (Au or Al). ${\text {n}}_m$ and $\kappa _{m}$ are the real and imaginary parts of the effective refractive index, $\theta _{m}$ is the incident angle of light, $d_m$ is the thickness, and $\phi _{m}$ is the phase delay of the optical wave in layer *m*. The manipulation of the $\phi _{m}$ by introducing a refractive index change is the key for dynamically tuning the resonance wavelength of the proposed FPNs. The term $\theta _{t}$ represents the transmitted wave angle; however, in this study, the incident angle for the optical study of all four structures is set to zero. Thus, the $\theta _{t}$ becomes zero, reducing the denominator of the $\phi _{m}$ equation to just $\lambda$. For the given four-layer systems, the *M* is written as:

12 $$\begin{aligned} M &= D^{-1}_1(D_{2}P_{2}D^{-1}_{2})(D_{3}P_{3}D^{-1}_{3})\nonumber \\{} &\qquad (D_{4}P_{4}D^{-1}_{4})D_{5}. \end{aligned}$$

Later, the reflection (*r*) and reflectance (*R*) are calculated using the $M_{21}$ and $M_{11}$ elements of *M*.

13 $$\begin{aligned} R = \mid r \mid ^2 = \Bigg |\frac{M_{21}}{M_{11}}\Bigg |^2 \end{aligned}$$

The results obtained using the TMM are dependent on the parameters n, $\kappa$, and *d* of the multilayer thin-film structure. For a given layer thickness (*d*), the values of n and $\kappa$ determine the *M*. In the absence of an applied potential, the effective refractive index values are constant across the semiconductor layer. However, when an external potential is applied, the effective refractive index values become different across the semiconductor layer, creating sublayers with different effective values of *d*, which leads to variations in *M* and hence, the *R*.

## Results and discussion

The electric potential and electron concentration distributions in the n-InSb and n-InAs layers of the four systems (as depicted in Fig. 3) were determined using the CHARGE solver of the Ansys LUMERICAL software [41]. The CHARGE solver utilizes material parameters from its built-in library, which includes the work functions of 5.1 eV for Au and 4.28 eV for Al and a relative dielectric permittivity of 80 for ${\text {TiO}}_2$. For the semiconductors InSb and InAs, the solver uses DC permittivities of 16.8 and 14.6, work functions of 4.71 eV and 5.08 eV, and intrinsic carrier densities of $1.92\times 10^{16}/\text {cm}^3$ and $7.24\times 10^{14}/\text {cm}^3$, respectively. The user can set the doping concentration of the semiconductor using the doping tool provided in the solver.

**Fig. 6:**
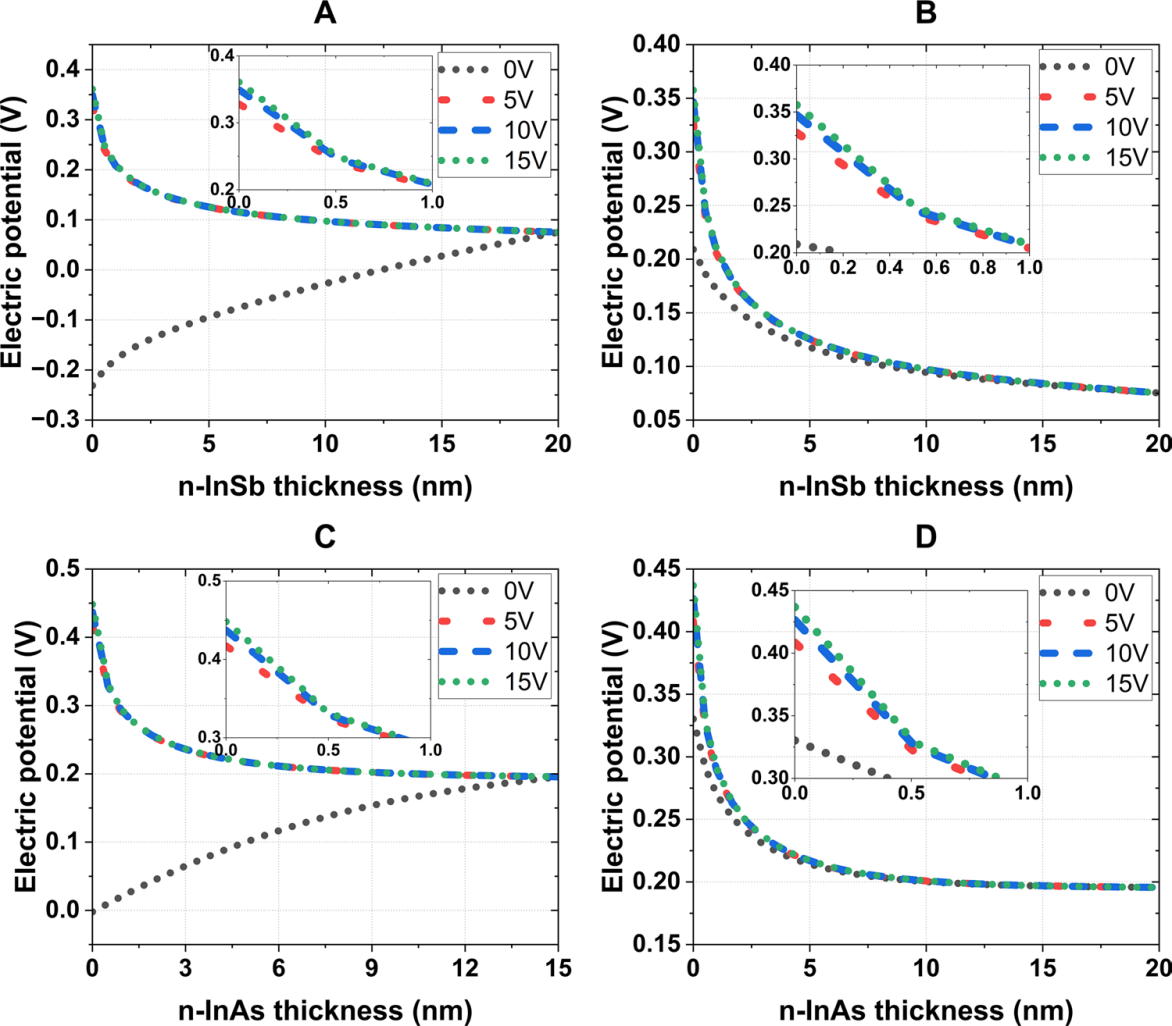
Electric potential ($\psi$) distribution in semiconductor layers of (**A**) Au (100 nm)–${\text {TiO}}_2$ (10 nm)–(n-InSb) (20 nm)–Au (17 nm), (**B**) Al (100 nm)–${\text {TiO}}_2$ (12 nm)–(n-InSb) (20 nm)–Al (8 nm), (**C**) Au (100 nm)–${\text {TiO}}_2$ (10 nm)–(n-InAs) (15 nm)–Au (15 nm), and (**D**) Al (100 nm)–${\text {TiO}}_2$ (15 nm)–(n-InAs) (20 nm)–Al (8 nm)

**Fig. 7:**
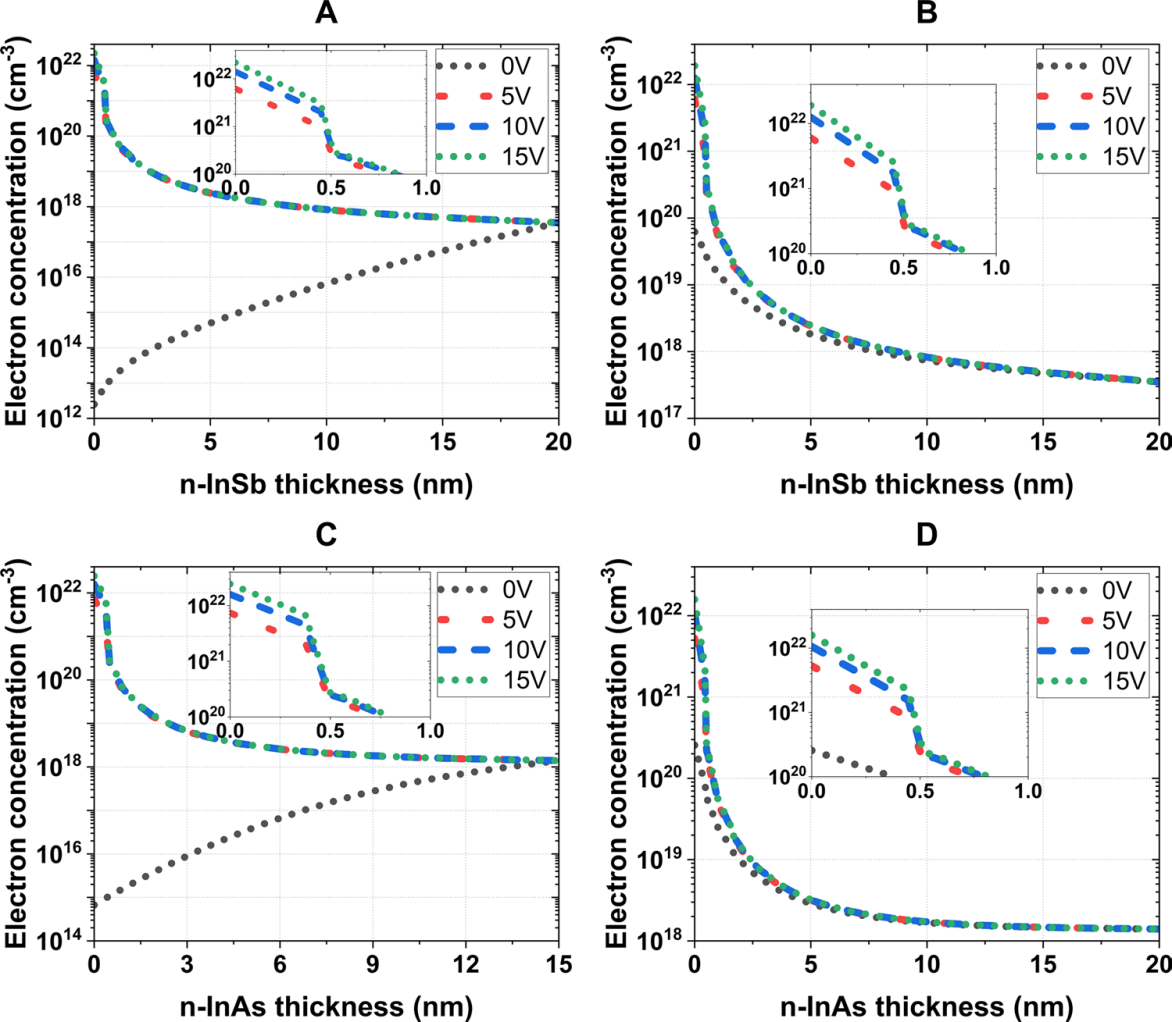
Electron distribution in semiconductor layers of (**A**) Au (100 nm)–${\text {TiO}}_2$ (10 nm)–(n-InSb) (20 nm)–Au (17 nm), (**B**) Al (100 nm)–${\text {TiO}}_2$ (12 nm)–(n-InSb) (20 nm)–Al (8 nm), (**C**) Au (100 nm)–${\text {TiO}}_2$ (10 nm)–(n-InAs) (15 nm)–Au (15 nm), and (**D**) Al (100 nm)–${\text {TiO}}_2$ (15 nm)–(n-InAs) (20 nm)–Al (8 nm)

The doping tool was set to n-type doping with constant concentrations of $3.5 \times 10^{17}$/cm$^3$ and $1.4 \times 10^{18}$/cm$^3$ for n-InSb and n-InAs, respectively. The solver uses Poisson’s equation to calculate the electric field, potential, and charge distribution across the semiconductor. For simulation, the gate metal layer (bottom metal layer) was simulated with 0 V, 5 V, 10 V, and 15 V with the top metal layer grounded. At 0 V, the observed electric potentials at the oxide-semiconductor interface of all four systems were due to the difference between the metal and semiconductor Fermi energy, resulting in a band bending.

As depicted in Fig. 6, a fraction of the total external voltage is dropped across the semiconductor layer with a $\psi _s$ value less than 0.5 V. With a 5 V increment, the $\psi _s$ value roughly increases by 0.02 V for all four systems. Figure 7 illustrates that the electron concentration at the interface can reach as high as $\sim$ $10^{22}/\text {cm}^3$ and exhibit an exponential decline across the semiconductor layer, eventually reaching the doped concentration of $3.5 \times 10^{17}$/cm$^3$ for n-InSb and $1.4 \times 10^{18}$/cm$^3$ for n-InAs near the edge of the semiconductor layer.

**Fig. 8:**
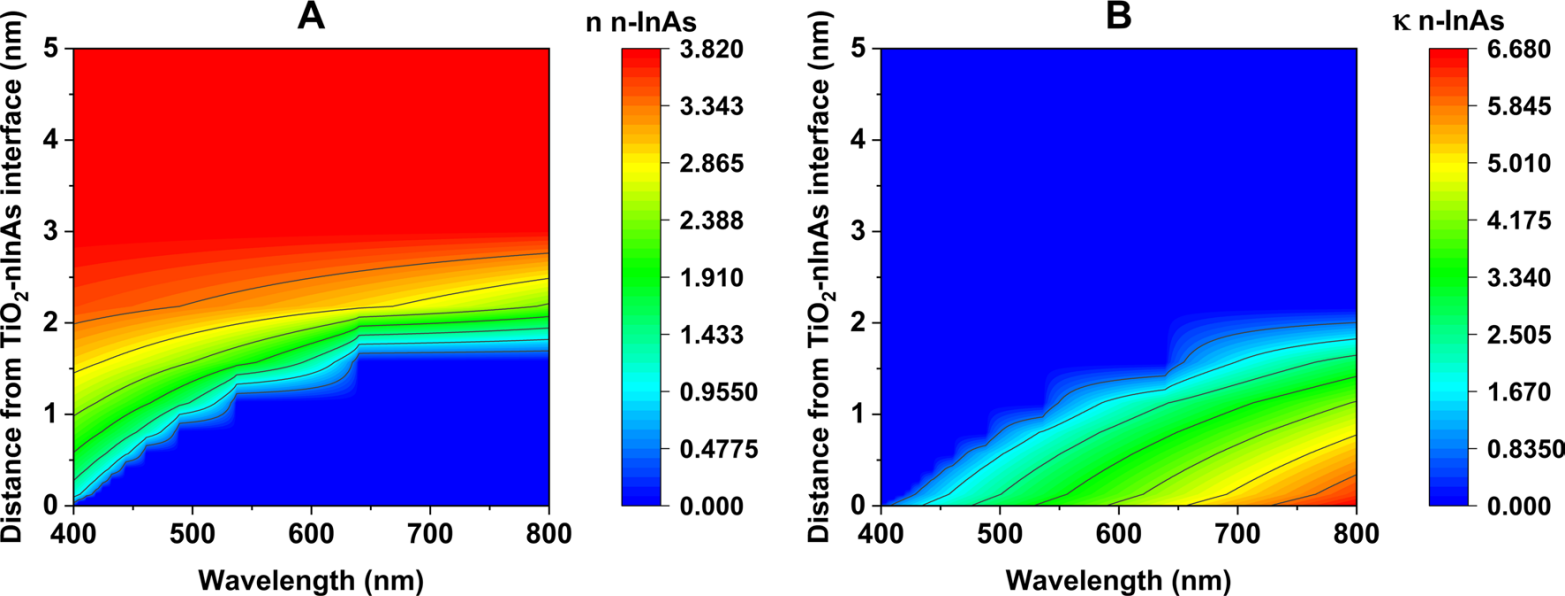
(**A**) Real and (**B**) imaginary refractive index gradient in n-InAs layer of Au–${\text {TiO}_2}$–(n-InAs)–Au structure at 10 V applied voltage

The calculated $\Delta$N across the semiconductor layer can be imported into the Ansys LUMERICAL software’s FDTD solver using the “np density” attribute [45]. The FDTD, a finite-difference time-domain routine then applies the Drude model to calculate the refractive index using the charge distribution from the np density over every 1-nm sublayer of the semiconductor. Figure 8 displays the gradient in the real and imaginary parts of the effective refractive index for the n-InAs layer of Au–${\text {TiO}}_2$–(n-InAs)–Au with a 10 V applied voltage (for the visible range), as calculated by the FDTD. Similar calculations are performed for all four systems at external voltages of 0, 5, 10, and 15 volts.

**Fig. 9:**
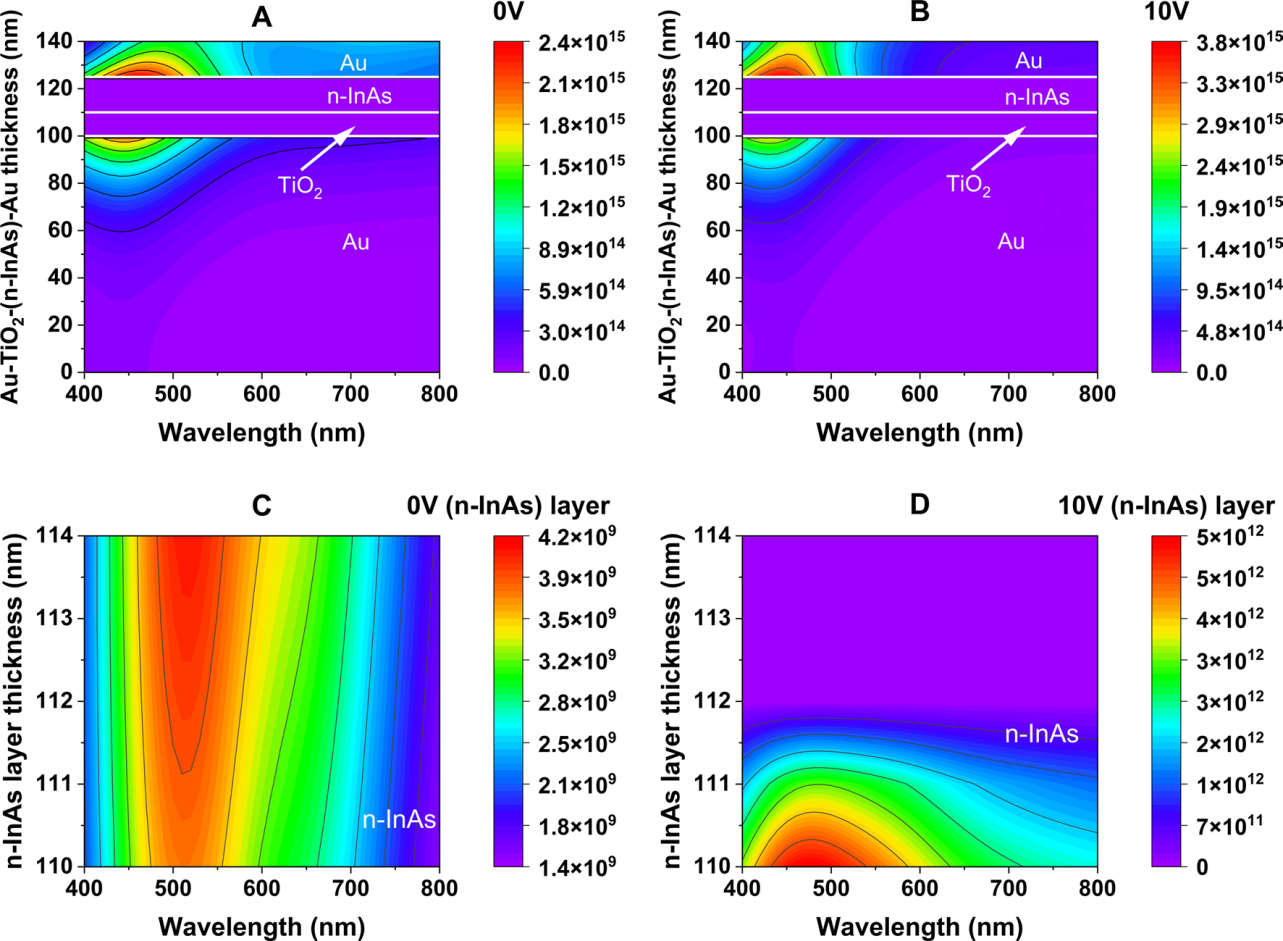
Absorbed power contour plot for (**A**) Au–${\text {TiO}}_2$–(n-InAs)–Au structure at 0 V, (**B**) Au–${\text {TiO}}_2$–(n-InAs)–Au structure at 10 V, (**C**) n-InAs layer from ${\text {TiO}_2}$-(n-InAs) interface of Au–${\text {TiO}}_2$–(n-InAs)–Au structure at 0 V, (**D**) n-InAs layer from ${\text {TiO}_2}$-(n-InAs) interface of Au–${\text {TiO}_2}$–(n-InAs)–Au structure at 10 V

As shown in Fig. 8, the refractive index near the ${\text {TiO}}_2$–(n-InAs) interface exhibits high $\kappa$ values and low n values. This implies that, under the influence of the applied voltage, up to 2 nm of the n-InAs material facing the ${\text {TiO}}_2$ layer should exhibit a different phase delay compared to the remaining part of the layer. To ascertain the optical impact of this change, the absorbed optical power profile is calculated using the FDTD [46]. The calculated absorption profile of the Au–${\text {TiO}}_2$-(n-InAs)-Au structure as a function of wavelength is presented in Fig. 9. It is evident that the metal layers absorb a majority of the incident light. However, the application of potential leads to a change in the power absorbed by the n-InAs layer closest to the ${\text {TiO}}_2$ layer for a depth up to 2 nm (Fig. 9D).

**Fig. 10:**
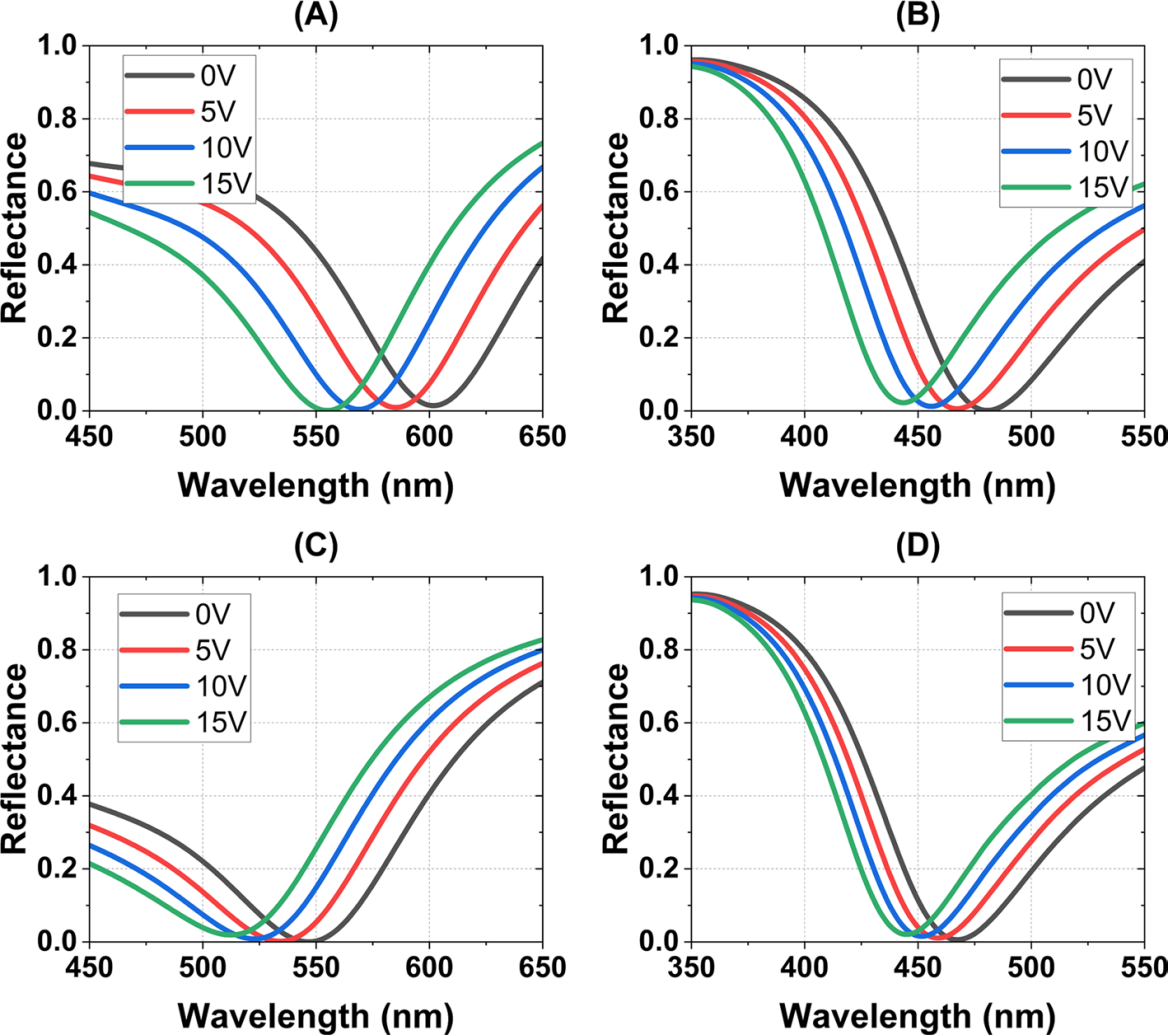
Reflectance spectrum of (**A**) Au (100 nm)–${\text {TiO}}_2$ (10 nm)–(n-InSb) (20 nm)–Au (17 nm), (**B**) Al (100 nm)–${\text {TiO}}_2$ (12 nm)–(n-InSb) (20 nm)–Al (8 nm), (**C**) Au (100 nm)–${\text {TiO}}_2$ (10 nm)–(n-InAs) (15 nm)–Au (15 nm), and (**D**) Al (100 nm)–${\text {TiO}}_2$ (15 nm)–(n-InAs) (20 nm)–Al (8 nm) for 0, 5, 10, and 15 volts applied potential

The optical impact of the refractive index gradient can be further elucidated by analyzing the reflectance spectrum of FPN structures. The TMM was utilized to calculate the reflectance spectrum of all four FPN structures under normal incidence. The refractive indices of Au, Al, and ${\text {TiO}_2}$ were obtained from previously published data by Gao et al. [47], McPeak et al. [48], and Sarkar et al. [49], respectively, for the transmission and propagation matrices. As previously mentioned, the refractive indices of n-InSb and n-InAs at various applied voltages were obtained using the Drude model in the FDTD index perturbation tool. The thickness of each layer for all four FPN structures used in TMM calculations is given in the introduction section of this article as well as in Fig. 3. The obtained reflectance spectra at different applied potentials for all four FPN structures under investigation using TMM are shown in Fig. 10.

At a potential of 0 V, the Au–(n-InSb) and Al–(n-InSb) structures exhibit perfect absorption at wavelengths of 602 nm and 480 nm, respectively. For the Au–(n-InAs) and Al–(n-InAs) structures, the corresponding wavelengths are 546 nm and 467 nm. By adjusting the thickness of the n-InSb and n-InAs layers in the structures, it is possible to shift the wavelengths of perfect absorption to longer wavelengths within the visible and infrared ranges. Application of a potential of 15 V to the structures results in spectral shifts of 47 nm, 37 nm, 33 nm, and 22 nm for the proposed Au–(n-InSb), Al–(n-InSb), Au–(n-InAs), and Al–(n-InAs) structures, respectively.

In the examined set of four structures, the Au–(n-InSb) combination produced the largest spectral shift. The use of Au as a reflective metal in FPNs caused a greater spectral shift compared to FPNs that employed Al. This effect can be attributed to the Fermi energy gap, also known as the flat-band potential, between the metal (Au or Al) and semiconductor (n-InSb or n-InAs) materials. The work function of Au (5.1 eV) is higher than that of n-InSb (4.71 eV) and n-InAs (5.08 eV). As a result, at 0 V applied potential, the depletion of holes arises at the oxide-semiconductor interface to maintain a constant Fermi energy between the metal and semiconductor. This hole depletion is created by the lack of electrons in that region (see Fig. 7A, C). Applying a potential of 15 V to the gate metal (Au) results in a significant increase in the number of accumulated electrons in n-InSb and n-InAs semiconductors. Specifically, in n-InSb, the number of accumulated electrons increases by a factor ranging from $10^{8}$ to $10^{10}$, while in n-InAs, the increase is a factor of $10^{6}$–$10^{7}$ (see Fig. 7A, C).

In FPNs that utilize Al (with a work function of 4.28 eV) as the gate metal, the work-function difference between the metal and semiconductor is of the opposite sign to that of FPNs with Au as the gate metal. As a result, at 0 V applied potential, the MOS structure generates a small accumulation of electrons at the oxide-semiconductor interface in order to maintain a constant Fermi energy. However, with the application of a positive potential to the gate metal, this accumulation increases by a factor of approximately $10^{2}$ in the first few nanometers of n-InSb and n-InAs (see Fig. 7B, D). Therefore, owing to the relatively greater change in electron concentration among the proposed structures, FPNs utilizing Au as the gate metal exhibit a higher spectral shift compared to those employing Al as the gate metal.

The limitation of the dynamic spectral shift, in either case, is determined by the potential limit, which is, in turn, determined by the breakdown voltage. It has been found that the breakdown strength of titanium dioxide is approximately $1.6\times 10^9$ V/cm [50]. Based on this, the MOS architectures proposed can withstand 16–24 V. The breakdown voltage can be enhanced by increasing the thickness of the oxide layer. However, any variation in the thickness of the oxide layer can have an effect on the optical response of the FPN.

## Conclusion

This study describes the development of dynamically tunable optical absorbers utilizing n-doped InSb and InAs as epsilon-near-zero (ENZ) semiconductors. The absorber structure models consist of nanoscale metal and semiconductor layers arranged in a metal-oxide-semiconductor–metal (MOSM) configuration. The MOSM architecture bears similarities to a MOS capacitor, which is effective in obtaining electrical control over charge distribution in the semiconductor layer. Optically, the MOSM structure serves as a Fabry–Perot nanocavity (FPN) that enables wavelength-selective light absorption. The selection of appropriate materials and thicknesses for each layer in the MOSM configuration provides perfect narrowband light absorption in the visible region, which renders the optical absorbers useful as color filters. The MOSM configuration is voltage-controlled, where the application of voltage results in charge accumulation in the n-InSb and n-InAs layers, thereby regulating the dielectric constant. The accumulated sublayers of n-InSb and n-InAs lead to a variation in the refractive index, which affects the effective phase delay of the semiconductor spacer. Such a phenomenon results in a change in the FPNs’ resonance in the visible range. The electro-optical analysis predicts that, with suitable materials and layer thicknesses, the MOSM structure can yield a reversible spectral shift of up to 47 nm at an applied potential of 15 V. The extent of the spectral shift in the absorbed color is influenced by both the applied voltage and the specific combination of metal and semiconductor materials employed. The study reveals that the proposed four MOSM combinations exhibit a spectral shift magnitude falling within the range of 22–47 nm.

## Data Availability

The datasets generated during and/or analyzed during the current study are available from the corresponding author on reasonable request.

## Abbreviations

ENZ: Epsilon-near-zero
FPN: Fabry–Perot nanocavity
MOSM: Metal-oxide-semiconductor–metal
MOScap: Metal-oxide-semiconductor capacitor
TMM: Transfer matrix method
FDTD: Finite-difference time-domain
